# Digestive dynamics: Unveiling interplay between the gut microbiota and the liver in macronutrient metabolism and hepatic metabolic health

**DOI:** 10.14814/phy2.16114

**Published:** 2024-06-17

**Authors:** Mrunmayee R. Kandalgaonkar, Virender Kumar, Matam Vijay‐Kumar

**Affiliations:** ^1^ Department of Physiology and Pharmacology University of Toledo College of Medicine and Life Sciences Toledo Ohio USA; ^2^ College of Pharmacy and Pharmaceutical Sciences University of Toledo Toledo Ohio USA

**Keywords:** bacterial metabolites, bile acids, bile salt hydrolase, short‐chain fatty acids

## Abstract

Although the liver is the largest metabolic organ in the body, it is not alone in functionality and is assisted by “an organ inside an organ,” the gut microbiota. This review attempts to shed light on the partnership between the liver and the gut microbiota in the metabolism of macronutrients (i.e., proteins, carbohydrates, and lipids). All nutrients absorbed by the small intestines are delivered to the liver for further metabolism. Undigested food that enters the colon is metabolized further by the gut microbiota that produces secondary metabolites, which are absorbed into portal circulation and reach the liver. These microbiota‐derived metabolites and co‐metabolites include ammonia, hydrogen sulfide, short‐chain fatty acids, secondary bile acids, and trimethylamine *N*‐oxide. Further, the liver produces several compounds, such as bile acids that can alter the gut microbial composition, which can in turn influence liver health. This review focuses on the metabolism of these microbiota metabolites and their influence on host physiology. Furthermore, the review briefly delineates the effect of the portosystemic shunt on the gut microbiota–liver axis, and current understanding of the treatments to target the gut microbiota–liver axis.

## 1 INTRODUCTION

### 1.1 The liver is an altruistic organ

Ancient Greeks considered *hepar*, or liver, to be the center of the soul. Greek mythology tells us that Prometheus created humans and gave them fire. This incurred Zeus' wrath, who ordered that Prometheus be chained and that every day an eagle would eat part of his liver. The liver would grow back overnight, and the eagle would return to its feast (van Gulik et al., 2018). This parable highlights the remarkable ability of the liver to regenerate into a whole, completely functional organ, which makes it a biological marvel in all vertebrates (Delgado‐Coello, 2021). Moreover, ancient Greeks considered the liver to be the seat of passion, which is evident from the liver's involvement in several vital bodily processes: it metabolizes all ingested xenobiotic compounds; it produces biomolecules required in digestion of food; and it influences metabolism in distant organs such as the bone, heart, brain, and the immune system (Cheng et al., 2021; Kalra et al.,^2023^).

Bile is one of the major products of the liver, produced about a liter per day. It is continuously produced by hepatocytes and stored in the gall bladder in most vertebrates and is released into the duodenum upon cue (Boyer, 2013; Hundt et al., 2022). The major components of bile are bile salts, cholesterol, and phospholipids—specifically, phosphatidylcholine. The bile mixes with the food in the duodenum and the bile salts emulsify ingested lipids, including fat‐soluble vitamins, aiding their assimilation. The portal vein carries absorbed nutrients to the liver where they are assimilated into building blocks of life. Further, the liver metabolizes all xenobiotic compounds such as orally ingested drugs using Cytochrome P450‐mediated hydroxylation and UDP‐glucanosyltransferases to increase their hydrophilicity; it converts simple molecules such as glucose and fatty acids into glycogen and triglycerides, respectively, for storage during positive energy balance. During fasting, the liver catabolizes glycogen and triglycerides via glycogenolysis and β‐oxidation, respectively, to generate ATP (Almazroo et al., 2017; Roumans et al., 2021; Rowland et al., 2013). The liver produces about 90% of all circulating proteins including albumin, fibronectin, angiotensinogen, and insulin‐like growth factors (Trefts et al., 2017). Further, it produces several enzymes such as alanine transaminase (ALT) and aspartate transaminase (AST), which play an important role in the biosynthesis of non‐essential amino acids via transamination (Gwaltney‐Brant, 2021; Ida & Washington, 2012) (Tables 1 and 2).

**TABLE 1:** List of abbreviations of analytes/metabolites.

Abbreviation	Expansion
UDP	uridine diphosphate
ATP	adenosine triphosphate
ALT	alanine transaminase
AST	aspartate transaminase
IgA	immunoglobulin A
SCFA	short‐chain fatty acids
H_2_	hydrogen
CO_2_	carbon dioxide
H_2_S	hydrogen sulfide
*Trp*	tryptophan
LPS	lipopolysaccharide
IL	interleukin
RSS	reactive sulfur species
ROS	reactive oxygen species
TGF‐β1	tumor growth factor β1
Nrf‐2	nuclear erythroid 2‐related factor 2
NF‐κB	nuclear factor κB
GPCR	G protein‐coupled receptors
TLR	toll‐like receptors
PPAR‐γ	peroxisome proliferator‐activated receptor gamma
JAK	Janus kinase
STAT	signal transducers and activators of transcription
MAPK	mitogen‐activated protein kinase
SAM	S‐adenosylmethionine
BA	bile acids
BSH	bile salt hydrolase
UDCA	ursodeoxycholic acid
TNFα	tumor necrosis factor alpha
TMAO	trimethylamine *N*‐oxide
TMA	trimethylamine
CYP7α1	cholesterol 7‐alpha‐hydroxylase
FXR	Farnesoid X receptor

**TABLE 2:** List of abbreviations of pathologies.

Abbreviation	Expansion
HE	hepatic encephalopathy
MAFLD	metabolic dysfunction‐associated fatty liver disease
MASH	metabolic dysfunction‐associated steatohepatitis
AH	alcoholic hepatitis
PSS	portosystemic shunt

The liver is considered a “first hit” moonlighting organ. It is endowed with ketone body biogenesis, but it lacks 3‐oxoacid CoA transferase, which prevents the liver from metabolizing ketone bodies as a source of energy. Nevertheless, it synthesizes them for use by other organs such as the brain and the heart during prolonged starvation, highlighting its altruistic nature (Rachel et al., 2020). Further, it acts like a “firewall”—it can detoxify microbial products and can mount appropriate immune responses through resident macrophages called Kupffer cells to capture any gut‐derived microorganisms that disseminate into portal circulation (Balmer et al., 2014; Wood, 2014). Further, the liver secretes copious amounts of acute phase proteins such as lipocalin‐2 and serum amyloid A, specifically during robust pro‐inflammatory response to regulate immune responses (Cai et al., 2016; Dahl et al., 2018). Thus, the liver is an epicenter for major metabolic pathways.

### 1.2 The gut microbiota is akin to an organ within an organ

The gut microbiota is a vast “organ within as organ” made up of 100 trillion microbes, belonging to bacteria, fungi, viruses, and more, making the human colon one of the most densely populated habitats on Earth. The gut microbiome carries about 3 million genes, approximately 130 times more than the host (Rinninella et al., 2019). Further, it shares of 37% of all metabolic reactions predicted to occur in the gut through their unique enzymes (Sridharan et al., 2014). Therefore, it is plausible that a healthy gut microbiome is imperative to maintaining good health.

Out of the 52 identified bacterial phyla, only 6–7 can colonize the human gut (King et al., 2019). Humans develop as fetuses in a “sterile” womb, although debatable (Kennedy et al., 2023; Perez‐Munoz et al., 2017; Stinson et al., 2019). Initial microbial colonization of newborn gut is very critical and has long‐lasting effects. For instance, the mode of delivery of a neonate greatly affects its gut microbial composition. During normal childbirth, the neonate is first exposed to the mother's vaginal canal—an opportunity for first colonization of the skin and the gut. Infants born through vaginal delivery inherit about 75% microbes from their mothers, whereas those born through cesarean section only host about 13% microbes the same as their mothers (Zhang et al., 2021). Next, when the child is fed breast milk, the gut is colonized by the human milk microbiota (Kalbermatter et al., 2021; Mueller et al., 2023; Nino et al., 2021; Rapin et al., 2023). Additionally, several other factors in the mother's milk are important for healthy gut microbiota colonization. For instance, immunoglobulin A (IgA) is important for microbial colonization and for maintaining a healthy gut microbiome by binding to opportunistic pathobionts in the gut aiding in their elimination through feces (Abokor et al., 2021; Saha et al., 2022).

**FIGURE 1:**
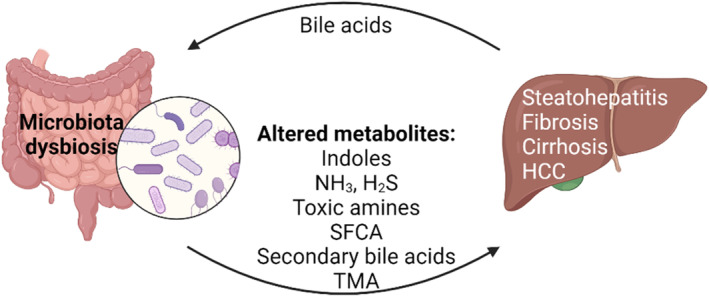
The gut microbiota and the liver partner to maintain optimal host physiology; defects in one cause malfunction of the other. Altered liver function affects the gut microbiota composition and gut dysbiosis influences liver functionality.

The gut microbiota and the liver appear to be unique centers of metabolism; however, they are not independent in functionality. For instance, while liver‐derived molecules regulate the gut microbiota composition, gut microbiota‐derived short‐chain fatty acids (SCFAs) influence liver metabolism (Cani, 2018). Thus, the gut microbiota and the liver regulate and assist each other to maintain optimal host physiology. The past decade has seen an explosion of research studying the gut microbiota–liver axis (8549 articles in the past 15 years on PubMed). This review summarizes key aspects of metabolism of macronutrients—proteins, carbohydrates, and lipids—that advances our understanding of the interaction between the gut microbiota and liver (Figure 1).

## 2 MACRONUTRIENT METABOLISM BY THE GUT MICROBIOTA AND THE LIVER

### 2.1 Proteins are digested by a combined effort from the gut microbiota

**FIGURE 2:**
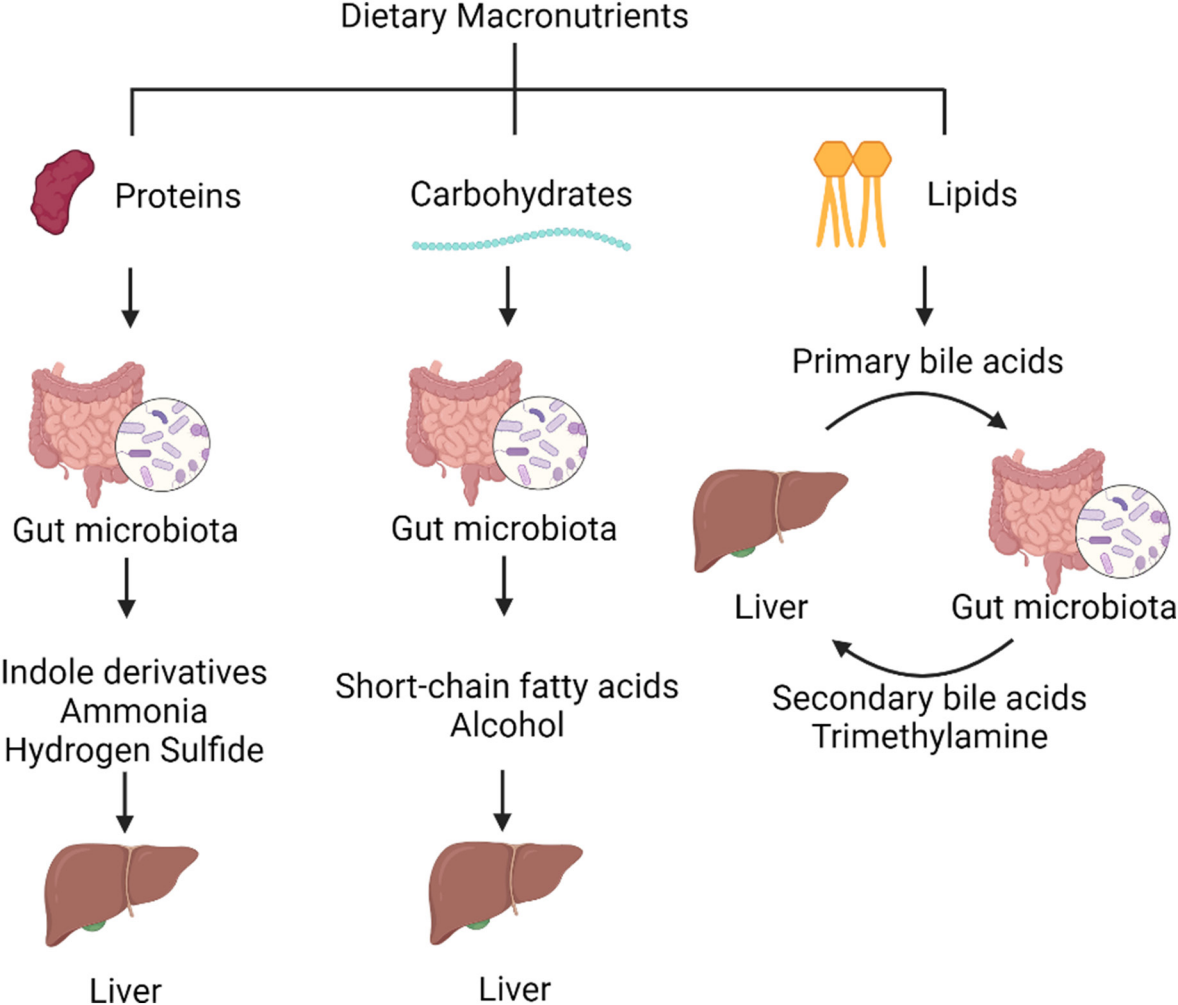
Major metabolites involved in the gut microbiota–liver axis. The gut microbiota is integral to macronutrient metabolism and produce a variety of secondary metabolites that can substantially influence liver metabolism and thus host physiology.

Over 90% of dietary proteins are digested and absorbed by the small intestines, whereas the rest is passed onto the colon. Due to the complexity of amino acid chemistry, any one species of gut microbiota, specifically bacteria, cannot metabolize all the different amino acids. Therefore, most bacteria cross feed off metabolites produced by other bacteria. Further, microbes use unabsorbed amino acids directly to bypass the need for specific amino acid biosynthesis. Clostridia spp. and Fusobacteria spp. are capable of assimilating proteins as carbon source. They can break down proteins to short‐ and branched‐chain fatty acids, ammonia, molecular H_2_, and CO_2_, alongside H_2_S, phenols, alcohols, and acids (Bartlett & Kleiner, 2022; Diether & Willing, 2019; Rodriguez‐Romero et al., 2022). Additionally, proteins can undergo putrefaction by the gut microbes generating toxic amines that promote colonic epithelial growth and have been implicated in colorectal cancers (Kaur et al., 2017; Nakamura et al., 2021) (Figure 2).

#### 2.1.1 Tryptophan metabolites

Tryptophan is an essential amino acid, and therefore, dietary intake of tryptophan is imperative to host metabolism. About 90% of the tryptophan is metabolized by the host via the kynurenine pathway, whereas only about 3% is metabolized to serotonin, and the rest is metabolized by the gut microbiota (Bosi et al., 2020). Tryptophan is thus one of the most important amino acids required for host homeostasis and gut microbiota eubiosis. Tryptophanase, which converts tryptophan into indole, is encoded in the *Trp* operon in microbes, out of which, *Escherichia coli* is the major producer of indole. Tryptophan is metabolized into indole and its derivatives, such as indole‐3‐propionate, skatole (methylindole), and indole‐3‐aldehyde, exclusively by the gut microbiota (Li, Zhang, et al., 2021). Indole and its derivatives act as endogenous ligands for the aryl hydrocarbon receptor (AhR). AhR is a nuclear receptor that resides in the cytoplasm in its inactive state. It binds to several types of ligands including xenobiotics, microbial products, and endogenous ligands, such as indole and its derivatives. For instance, supplementation of indole alleviates lipopolysaccharide (LPS)‐induced hepatitis in mice (Beaumont et al., 2018; Wang et al., 2022). Further, pro‐ and prebiotic supplementation increases AhR activity (Wu et al., 2022).

In addition to *E. coli, Lactobacillus reuteri*, a commensal gut bacterium, possesses several enzymes required for tryptophan catabolism to indole and indole‐derivatives. *L. reuteri*‐colonized mice have greater circulating indole derivatives that activate AhR, prompting NF‐κB suppression and increasing IL‐17 and IL‐22 production (Montgomery et al., 2022; Pernomian et al., 2020).

In the liver, indole is further metabolized into indoxyl‐3‐sulfate by cytochrome P450 enzymes such as Cyp2E1, followed by sulfotransferase (Hendrikx & Schnabl, 2019). Indoxyl‐3‐sulfate is a potent uremic toxin that causes kidney damage, and interestingly, increases hepatic multidrug resistance protein 1 through AhR signaling (Santana Machado et al., 2018). Liver diseases such as alcoholic steatohepatitis, fibrosis, and cirrhosis are marked with diminished AhR expression (Carambia & Schuran, 2021; Patil et al., 2023).

#### 2.1.2 Ammonia

Amino acid catabolism by gut microbes, such as *Clostridia*, *E* *nterobacteria*, and *Bacillus* spp., can lead to the generation of ammonia, which is used to generate glutamine in the gut, and is absorbed into portal circulation (Vince & Burridge, 1980). Ammonia is transported in systemic circulation by glutamate as glutamine, a reaction catalyzed by hepatic glutamine synthase. However, acute or chronic liver diseases can hamper the liver's capacity to metabolize ammonia and release it in systemic circulation. Being a small molecule, ammonia can easily cross the blood–brain barrier. Brain cells are efficient in integrating ammonia into glutamate to form glutamine. However, excess of ammonia can cause depletion of glutamate in brain cells. This is overcome by an anaplerotic reaction that is converting α‐ketoglutarate into glutamate, but at the cost of α‐ketoglutarate depletion. Thus, ammonia indirectly attenuates the TCA cycle, often leading to hepatic encephalopathy (HE). Furthermore, ammonia can directly inhibit enzymes such as pyruvate dehydrogenase and α‐ketoglutarate dehydrogenase that can lead to excessive lactate and pyruvate accumulation in the brain. Patients with advanced stages of liver cirrhosis often present with signs of HE, such as hepatic coma, and greater serum lactate and pyruvate (Aldridge et al., 2015; Deutsch‐Link et al., 2022; Jayakumar & Norenberg, 2018).

Increasing glutamine consumption elevates α‐ketoglutarate that stimulates mammalian target of rapamycin (mTOR) activity, which leads to aerobic glycolysis in the cell. This can cause dramatic effects in cancer patients as mTOR is known to promote cancer cell growth (Albrecht et al., 2010; Holecek, 2013; Li, Zhu, et al., 2021; Yoo et al., 2020). Despite these side effects, short‐term glutamine supplementation is shown to maintain a healthy gut microbiota by increasing the production of secretory IgA in the liver and intestines, which allows for selection of eubiotic gut microbiota and elimination of pathogenic microbes, improving several conditions such as hypertension (Abokor et al., 2021; Perna et al., 2019; Saha et al., 2022). Therefore, it is imperative to determine whether glutamine supplementation is essential, per individual, to weigh its cons against its pros.

#### 2.1.3 Hydrogen sulfide

The liver produces Hydrogen sulfide (H_2_S) essential for optimal lipid metabolism and growth of several hepatic cells. H_2_S promotes lipid oxidation, and therefore prevents hepatic steatosis, and metabolic dysfunction‐associated fatty liver disease (MAFLD) and metabolic dysfunction‐associated steatohepatitis (MASH) (Norris et al., 2011). Gut bacteria, such as the genus *Desulfovibrio*, produce H_2_S during metabolism of sulfur‐containing amino acids such as methionine, cysteine, and taurine (Carbonero et al., 2012; Murros, 2022; Wolf et al., 2022). H_2_S is a reactive sulfur species (RSS) that prevents oxidative stress by increasing γ‐glutamylcysteine synthetase activity and therefore, glutathione. Additionally, H_2_S can directly scavenge reactive oxygen species (ROS) (Xiao et al., 2018). Further, H_2_S increases glutathione production, which inhibits ferroptosis by suppressing tumor growth factor β 1 signaling and stimulating nuclear erythroid 2‐related factor 2 (Nrf‐2) to alleviate acute liver failure (He et al., 2023; Huang et al., 2022; Kimura et al., 2010; Wang et al., 2023; Zhang, Jiang, et al., 2023). Intriguingly, H_2_S produced by dysbiotic gut microbes such as *Escherichia*, *Klebsiella*, *Proteus*, and *Fusobacteria* stimulates proliferation and activation of hepatic stellate cells, and their transformation to fibroblast‐like cells, increasing collagen deposition in the liver, leading to liver fibrosis, and eventually liver cancer (Chirindoth & Cancarevic, 2021; Damba et al., 2019; Liu et al., 2022). Exogenous supplementation of H_2_S donors, such as GYY4137, is a therapeutic against hyperlipidemia‐associated liver injury (Zhao, Song, et al., 2021). Interestingly, Lachnospiraceae and Ruminococcaceae spp. convert cysteine into cysteine persulfide, an RSS, using pyridoxal 5′‐phosphate‐dependent sulfurtransferases that can alleviate liver damage (Ida et al., 2014; Uchiyama et al., 2022). H_2_S, therefore, acts as a double‐edged sword in the pathogenesis of liver diseases.

### 2.2 Gut microbiota supplements vital enzymes for carbohydrate metabolism

Carbohydrates are the preferred source of carbon for most living organisms, including gut microbes. Our diet contains two types of carbohydrates: simple sugars and dietary fibers. While sugars are easily digested in the small intestines, dietary fiber is metabolized by the gut bacteria in the colon. Further, dietary fiber can be divided into water‐insoluble and ‐soluble fiber. Water‐insoluble fiber is majorly cellulose, and forms the roughage of the fecal matter and holds water to maintain gut motility and consistency of the feces. On the other hand, water‐soluble (fermentable) fiber is actively metabolized into simpler carbohydrates. Humans are deficient in enzymes known as carbohydrate‐active enzymes (CAZy) that digest fibers (Guan et al., 2021; Parmeshwar Vitthal Gavande & Fontes, 2023). These CAZy are supplemented by the gut microbiota. The major by‐products of fermentable fibers and gut microbiota include SCFAs and alcohols (Figure 2).

#### 2.2.1 Short‐chain fatty acids

Acetate, propionate, and butyrate account for more than 95% of the SCFA produced in the gut (den Besten et al., 2013). SCFAs, specifically butyrate, are mainly used by intestinal epithelia as a Carbon source. Additionally, excess SCFAs are absorbed in portal circulation and used as anabolic raw materials in the liver; propionate is used in gluconeogenesis, whereas acetate and butyrate are used for lipogenesis. SCFAs interact with several microRNAs (miRNA) involved in metabolism and cell cycle regulation and therefore cancer. miRNA mir‐33b, among others, increases β‐oxidation of fatty acids, reduces hepatic lipogenesis, and reduces the risk of steatosis, and therefore MAFLD (Fernandez‐Hernando et al., 2011). Thus, it aids in correcting obesity and diabetes by increasing insulin sensitivity. Further, SCFAs are anti‐inflammatory and can suppress nuclear factor κB (NF‐κB) either by antagonizing G protein‐coupled receptors or toll‐like receptors, or by inhibiting histone modifications by suppressing histone deacetylase and stimulating peroxisome proliferator‐activated receptor gamma (PPAR‐γ) (Amiri et al., 2022; Cani, 2018; Chen et al., 2023; Fernandez‐Hernando et al., 2011; Hu et al., 2011; Prins et al., 2021; Xu et al., 2022; Zhao, Wang, et al., 2021). Acetate produced by *Bifidobacterium pseudolongum* suppresses cell cycle and drives apoptosis by suppressing IL6/JAK/STAT signaling, thus inhibiting the progression of MAFLD to liver cancer (Song et al., 2023). Additionally, acetate inhibits the production of IL‐17A by type 3 innate lymphoid cells in liver and prevents HCC progression (Hu et al., 2023). Propionate is known to alleviate liver injury by hindering NF‐κB signaling and suppressing inflammation (Chen et al., 2023). Furthermore, butyrate produced by *Clostridia* spp. induce colonic regulatory T cells in the colon, with implication in pathogenesis of inflammatory bowel diseases (Deleu et al., 2021). Interestingly, administration of SCFA cocktail increases the regulatory T‐cell population in the blood and causes immunosuppression and broadly serve as anti‐inflammatory (Huang et al., 2023). Therefore, SCFA are critical metabolites produced by the gut microbiota that dictate several aspects of liver health.

#### 2.2.2 Alcohol

The gut microbiota produces alcohol upon carbohydrate fermentation. The liver converts it into SCFA or into an aldehyde. Abundant Proteobacteria and high‐alcohol‐producing *Klebsiella pneumoniae* in the gut microbiota are a sign of dysbiosis and are often reported in patients with inflammatory bowel disease; they are also more prone to developing MAFLD (Andoh & Nishida, 2023; Li, Li, et al., 2021; Zhang, Su, et al., 2023). Further, microbiota can increase the severity of alcoholic hepatitis (AH) caused by alcohol consumption. For instance, fecal abundance of *Enterococcus faecalis* positively correlates to severity of AH and results in the worst prognosis. *E. faecalis* secretes a two‐subunit exotoxin, cytolysin, which causes hepatocyte death and liver injury. Intriguingly, targeting *E. faecalis* with a bacteriophage of the Picovirinae group in a humanized‐mouse model reduced hepatic cytolysin and prevented alcohol‐induced liver damage (Duan et al., 2019). Similarly, fecal abundance of *C. albicans* is higher in AH patients; it secretes a cytolytic peptide candidalysin, an exotoxin that damages epithelial mucosa and induces inflammatory cytokines such as interleukins IL‐1α, IL‐1β, and IL‐8, through *c*‐Fos and mitogen‐activated protein kinase (MAPK) signaling and aggravates alcohol‐associated liver damage (Chu et al., 2020).

In another mechanism, chronic alcohol consumption may lead to gut microbiota alterations, particularly bacterial overgrowth, which disrupts the intestinal barrier, causing dissemination of microbial products to the liver via portal circulation, leading to liver injury and exacerbating disease progression (Chen et al., 2015). Furthermore, alcohol consumption disrupts normal methionine metabolism in the liver by inhibiting methionine synthase, which increases homocysteine and decreases *S*‐adenosylmethionine (SAM), a universal methyl group donor for numerous critical biochemical reactions. *Saccharomyces cerevisiae*, the common gut yeast, enhances hepatic SAM and protects against alcohol‐induced liver damage (Izu et al., 2006). Thus, even though alcohol abuse is associated with gut microbiota dysbiosis (Chen et al., 2022), the gut microbes produce alcohol as a by‐product of carbohydrate metabolism, with profound effects on liver health.

### 2.3 Lipid absorption is regulated by the gut microbiota composition

Dietary lipids majorly include triglycerides with smaller quantities of phospholipids, sphingolipids, cholesterol, and lipid‐soluble vitamins (Iqbal & Hussain, 2009; Jian et al., 2022). Lipids are majorly assimilated in the small intestine with the aid of liver‐derived bile salts. The direct involvement of gut microbiota in lipid digestion was debated; however, in a recent elegant study, Li et al showed that *Oscillobacter* spp. in the gut is capable of cholesterol metabolism through dehydrogenation and glycosylation (Li et al., 2024). Additionally, the role of gut microbes in regulating lipid absorption is well established. For instance, *Bacteroides thetaiotaomicron* is associated with higher plasma cholesterol and cholesterol sulfates (Le et al., 2022). Further, dietary lipids influence gut microbial composition, and therefore, affect microbiota‐derived secondary metabolites (Schoeler & Caesar, 2019) (Figure 2).

#### 2.3.1 Bile acids

Primary bile acids (BA) are produced by the liver from cholesterol and stored in the gall bladder, in most vertebrates, and are associated with phospholipid micelles. They are produced by hepatocytes conjugated to predominantly taurine in rodents or glycine in humans. Dietary lipids stimulate the secretion of cholecystokinin that contracts the gall bladder. Over 95% of BAs released in the duodenum are eventually reabsorbed in the ileum and recycled via enterohepatic circulation. The rest of the BAs enter the colon where microbes, specifically bacteria deconjugate primary BA from taurine or glycine via bile salt hydrolase (BSH) and then convert them into secondary BAs such as deoxycholate and lithocholate by a series of bacteria‐mediated dehydroxylation, epimerization, and dehydrogenation reactions (Ridlon et al., 2014; Zeng et al., 2019). Recently, Rimal et al. and Guzior et al. showed that BSH also has *N*‐acyltransferase activity that allows it to conjugate amines to BAs, forming bacterial bile acid amidates, also known as microbially conjugated BAs (Guzior et al., 2024; Rimal et al., 2024). However, the biological significance of these BAs are yet to be identified.

Another class of BAs, called allo‐BAs, are implicated in liver and colorectal carcinogenesis (Mendoza et al., 2003; Tadano et al., 2007). These BAs carry a hydrogen or a hydroxyl group in α‐position on C‐5 of the sterol nucleus, making the BA “flat”, as opposed to “kinked” caused by the group at C‐5 in the β‐position. Allo‐BAs are fetal BAs, diminished after birth, but reappear during liver regeneration and liver cancer, analogous to α‐fetoprotein (Mendoza et al., 2003). Further, they are potent cholestatic agents but their role in post‐natal liver is not understood (Vore et al., 1989). Gut bacteria such as *Firmicutes* and *Bacteroidetes* spp. convert keto‐BAs (e.g. 3‐oxo‐lithocholic acid) into keto allo‐BAs (e.g., 3‐oxo‐allolithocholate) by 3‐oxo‐Δ^4^‐5ɑ‐reductases encoded by the BA inducible *baiP*/*baiJ* genes, or to allo‐BAs (e.g., allolithocholate) (Lee et al., 2022). Interestingly, 3‐oxo‐lithocholate is shown to reduce Th_17_ differentiation, whereas allolithocholate increased T_reg_ differentiation by increasing mitochondrial ROS and suppressing inflammation in the gut through the nuclear hormone receptor NR4A1. Patients with inflammatory bowel diseases often have diminished serum isoallolithocholate, highlighting its importance in immune regulation (Hang et al., 2019; Li, Hang, et al., 2021).

Bile constituents play an important role in maintaining gut eubiosis. Bile consists of BA and cholesterol, both conjugated to phospholipid micelles. Excessive BA in the bile can cause liver injury as well as gut microbiota dysbiosis by forcing a switch to microbes that can metabolize the altered BA pool (Liu, Yang, et al., 2021; Stacy et al., 2021; Tsuei et al., 2014; Wang et al., 2019). Though secondary BA are harmless at physiological levels, excess in fact causes damage to multiple organs. For instance, exogenous administration of deoxycholate and lithocholate increases liver injury as reflected in increased serum ALT, cellular necrosis, and ductular reaction in rodents (Delzenne et al., 1992; Song et al., 2011). Furthermore, excessive secondary BA in the gut can lead to gut dysbiosis, exacerbating liver injury by increasing gut permeability and allowing LPS leakage into portal circulation causing metabolic endotoxemia (Zhang et al., 2022). LPS is an endotoxin present on Gram‐negative bacteria and can lead to liver injury as severe as acute liver failure (Jiang et al., 2018). Mice and humans with post‐cholecystectomy diarrhea have greater secondary BA than primary BAs in their gut due to a dysbiotic gut microbiota with more abundant *Clostridia* and *Lachnospiraceae* spp. that have 7α‐dehydroxylase activity (Xu et al., 2023). On the other hand, hydrophilic BA such as ursodeoxycholic acid (UDCA) and its derivatives promote BA hydrophilicity, inhibit the production of TNFα and thus suppress inflammation, reversing hepatic injury (Buryova et al., 2013; Cabrera et al., 2019) and are used as a first line of drug to treat several liver diseases including cholelithiasis (Hall et al., 2023; Robles‐Diaz et al., 2021). BA, therefore, play a vital role in lipid metabolism and regulation of gut microbiota eubiosis.

#### 2.3.2 Trimethylamine *N*‐oxide

Trimethylamine *N*‐oxide (TMAO) is a by‐product of phospholipid metabolism by the gut microbiota. Gut microbes metabolize lipid moieties such as choline in phosphatidylcholine, along with L‐carnitine, and betaine into trimethylamine (TMA) using TMA lyases, cutC/D, cntA/B, and yeaW/X (Cai et al., 2022). TMA is absorbed into portal circulation and further metabolized to TMAO by host hepatic flavin monooxygenases 1 and 3. High TMAO levels are associated with several pathological conditions, including cardiovascular and gastrointestinal diseases, and is emerging as a novel biomarker of diseases (Gatarek & Kaluzna‐Czaplinska, 2021; Janeiro et al., 2018; Li et al., 2022; Zhen et al., 2023). It is yet unknown what gut microbiota families produce TMA; however, recent studies have highlighted certain phyla such as *Firmicutes* and *Proteobacteria* that are associated with the TMA production in the presence of choline in the gut (Liu & Dai, 2020; Rath et al., 2017). Since choline is also produced by host hepatocytes, excessive TMAO production can mimic choline deficiency and can lead to liver diseases such as MAFLD (Dumas et al., 2006). Furthermore, TMAO can disrupt the BA pool by suppressing bile acid transporters such as organic anion transporter in the gut and cholesterol 7‐α‐hydroxylase (CYP7α1) (Theofilis et al., 2022). Reducing the gut BA pool can negatively impact emulsification and absorption of dietary lipids and therefore may cause steatorrhea and lipid‐soluble vitamin deficiencies. These studies highlight the importance of dietary lipids and their role in maintaining gut microbiota eubiosis.

### 2.4 Portosystemic shunt allows portal blood to bypass the liver

Portosystemic shunt (PSS) refers to a vascular anomaly that connects portal circulation directly to systemic circulation, bypassing the liver. It may be congenital, or surgically created to alleviate portal hypertension in cirrhotic patients. Intriguingly, some liver cirrhosis patients develop spontaneous PSS and exhibit hyperammonemia and HE (Jayakumar & Norenberg, 2018; Lv et al., 2022; Yeoh et al., 2022, 2023). Since the liver is bypassed in the presence of PSS, it is interesting to speculate unprocessed diet and gut microbe‐derived metabolites in the circulation. Further, presence of PSS spills bile acids reabsorbed enterohepatically from portal circulation into systemic circulation, increasing the systemic BA pool (Yeoh et al., 2022). Further, in the presence of PSS, gut microbiota‐derived TMA accumulates in the blood and is excreted by the kidneys. PSS patients often suffer from “fishy urine” and “fishy sweat,” which occurs due to excessive excretion of TMA with reduced hepatic conversion of TMA to odorless TMAO (Landfald et al., 2017; Mackay et al., 2011). Further, heightened TMA levels in the gut can disrupt tight junctions between the intestinal epithelia and increase gut permeability, and exacerbate LPS‐induced liver injury (Yan et al., 2023).

Studies from our lab demonstrate that 10%–12% of the C57BL/6J mice, a widely used animal model in biomedical research, display congenital PSS and suffer from cholemia, a clinical condition associated with excess of BAs in systemic circulation. Furthermore, PSS mice suffer from steatorrhea, and vitamins A and D and essential fatty acid deficiency (Yeoh et al., 2022). Mice with congenital PSS are highly susceptible to cholestatic liver cancer when fed diets containing inulin, a plant‐derived fermentable fiber. Further, mice deficient in AhR have 100% incidence of PSS (Yeoh et al., 2023). Thus, PSS underscores the importance of connectivity between gut, gut microbiota, and liver.

## 3 TARGETING THE GUT–LIVER AXIS

As described above, the gut microbiota plays a crucial role in maintaining health, especially liver health. Gut microbes produce metabolites that aid or harm the liver and affect its function. Recent studies have shown that correcting dysbiosis can reverse or alleviate conditions as severe as hypertension (Mei et al., 2022). Several clinical trials have targeted the gut microbiota to correct liver disorders (Wiest et al., 2017).

**FIGURE 3:**
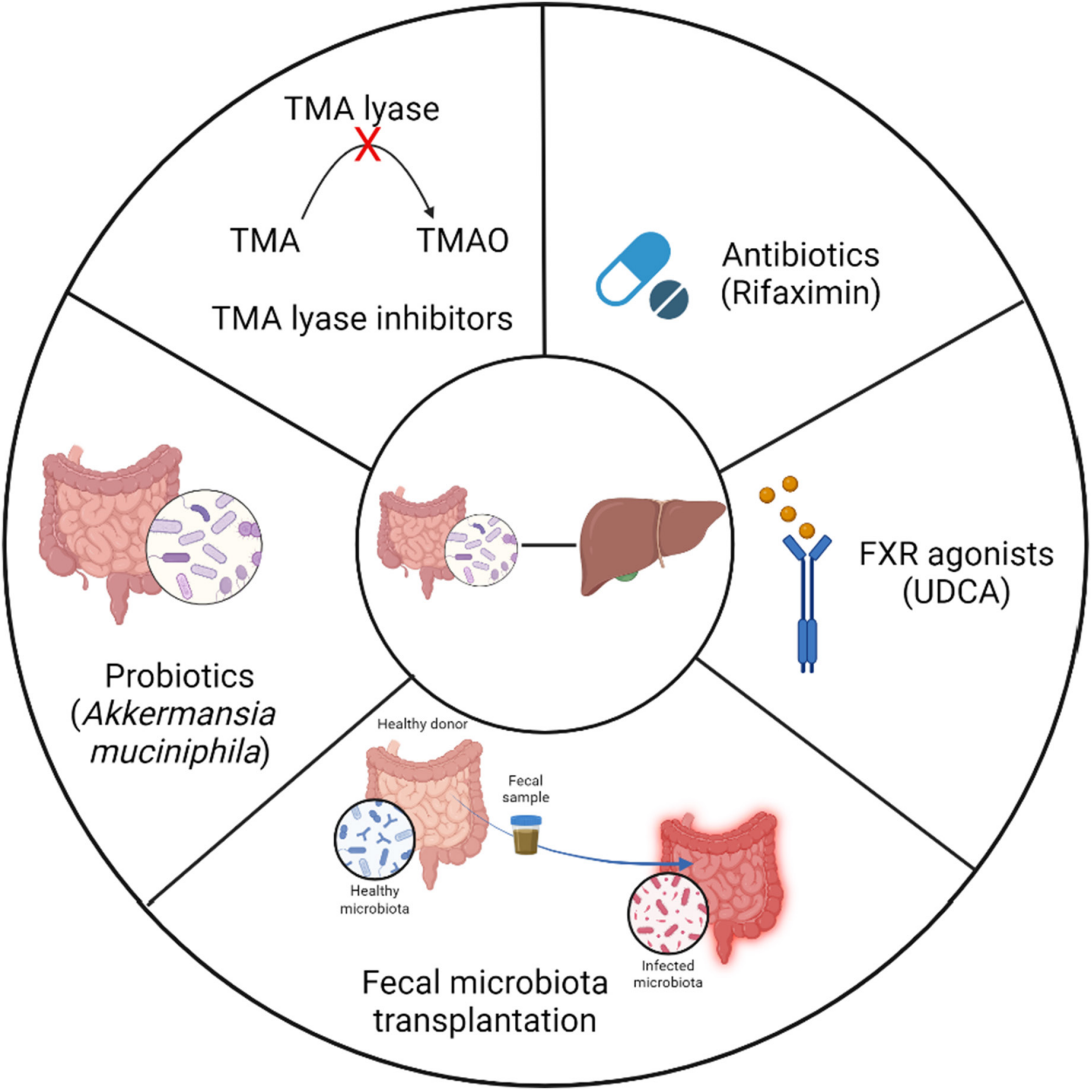
Potential targets for intervening gut microbiota and liver axis.

BA produced by the host has a profound impact on gut microbial composition. Activating FXR in the gut using agonist such as obeticholic acid and UDCA are effective in improving the BA composition and alleviating MAFLD (Friedman et al., 2018; Ubeda et al., 2016). Antibiotics allow for either elimination of pathogenic bacteria or complete elimination of gut bacteria, followed by recolonization with eubiotic microbes. Further, bacteria such as *Enterobacteriaceae*, *Enterococcaceae*, and *Streptococcaceae* are associated with liver cirrhosis (Li, Mao, et al., 2021). Interestingly, rifaximin, a broad‐spectrum antibiotic has shown to improve symptoms of liver cirrhosis, and HE by correcting dysbiosis (Caraceni et al., 2021; Israelsen et al., 2023; Mendez‐Sanchez et al., 2023; Yu et al., 2021; Zacharias et al., 2023) (Figure 3).

The most efficient procedure for correcting gut dysbiosis, including elimination of potent gut pathogen *Clostridium difficile*, is fecal microbiota transplantation (FMT). It involves transplantation of feces from a healthy donor to a receiver with dysbiosis (Gupta et al., 2016; Shasthry, 2020). This procedure is used to treat conditions such as inflammatory bowel diseases (Kedia et al., 2022; Tan et al., 2020), and has shown to correct dysbiosis, increase SCFA production (Su et al., 2022; Zhang, Jian, et al., 2023), restore normal BA composition, and alleviate liver injury in mice and humans (Bustamante et al., 2022; Hu et al., 2022; Liu, Fan, et al., 2021; Ramos et al., 2022; Weingarden et al., 2014; Xue et al., 2022).

To avoid the risks associated with FMT, several studies have proposed using isolated eubiotic microbes from healthy hosts. For instance, Celiberto et al. isolated commensal bacteria such as *Lactobacillus* and *Bifidobacterium* spp. from healthy C57BL/6 mice, and administered them to colitic C57BL/6 mice to alleviate colitis (Celiberto et al., 2018). Moreover, administration of a mixture of *Lactobacillus* and *Bifidobacterium* strains along with the prebiotic oligofructose has been shown to reduce liver injury and improve liver functions (Ma et al., 2013). Similarly, oral administration of *Akkermansia muciniphila* improved gut barrier and reduced dyslipidemia in mice suffering from metabolic syndrome (Plovier et al., 2017). Furthermore, a bacteriophage to target certain bacteria, such as the high‐alcohol‐producing *K. pneumoniae* and *E. faecalis* associated with AH, is shown to reduce their abundance in the dysbiotic gut, alleviating alcoholic liver disease (Duan et al., 2019; Gan et al., 2023).

In addition to modulating gut microbiota, their secondary metabolites can be targeted. TMA, the precursor of TMAO, is produced by the gut microbiota using TMA lyases that can be inhibited using small molecule inhibitors, indomethylcholine and fluoromethylcholine. Such inhibition of TMA lyases has shown to reduce TMA production in the gut, and subsequent production of TMAO, alleviating AH (Helsley et al., 2022). Further, AhR recognizes a plethora of ligands of physiologic, microbial, and xenobiotic origin and is a promising candidate for treating liver diseases associated with dysbiosis. Mice deficient in AhR in intestinal epithelia suffer from severe alcoholic liver disease and gut dysbiosis when fed an ethanol diet. However, when C56BL/6 were fed an ethanol diet along with synthetic AhR agonists, the mice were protected from alcoholic liver disease (Qian et al., 2022) (Figure 3).

The accumulated gut microbiome investigations highlight the importance of in‐depth basic research. Further, it warrants more sensitive and sophisticated techniques to identify abundance, or reduced levels of bacteria at the species level. Deprivation or accumulation of their unique metabolite(s) in various pathophysiological conditions may aid in designing novel therapeutics against gut microbiota dysbiosis‐associated diseases.

## 4 CONCLUSION

The liver and the gut microbiota constantly engage in a bidirectional relationship that benefits one another. This symbiosis, however, is often perturbed by liver pathologies such as gut microbiota dysbiosis, MAFLD, cirrhosis, and portosystemic shunting. The consequent impairment of hepatic function can lead to a change in bile acid levels or their profiles and substantial alterations in gut microbial composition. This can substantially alter the gut microbiota‐derived secondary metabolite homeostasis and worsen liver damage. Accordingly, a prevailing hypothesis had gained traction over the recent decades, is that correcting dysbiosis can increase production of beneficial metabolites and alleviate liver injury.

Although many studies acknowledge that having a healthy microbiota is important for health, the composition of such a “perfect microbiome” remains elusive and likely to be personalized for every individual. Such notion epitomizes the concept of personalized gut microbiome medicine to treat various diseases. Gut microbiota could be modulated by fecal microbiota transplant, intake of probiotics and prebiotics, and changing dietary habits. The scope of personalized gut microbiota and modified diet is to ensure eubiosis in the gut, and therefore, a better quality of life. Nevertheless, recognizing the complex interaction between the gut microbiota and the metabolism of their metabolites by the liver is a similarly critical yet understudied domain to develop treatments for liver disease.

## AUTHOR CONTRIBUTIONS

M.R.K. drafted and prepared the manuscript. V.K. and M.V.K. edited and contributed scientific prospects to the manuscript.

## FUNDING INFORMATION

M.V.K. is supported by the National Institutes of Health grants RO1 CA219144 and RO1 DK134053.

## CONFLICT OF INTEREST STATEMENT

The authors declare that they have no competing interests.

## ETHICS STATEMENT

None.

## Data Availability

Not applicable.
